# Tamoxifen Activates Dormant Primordial Follicles in Mouse Ovaries

**DOI:** 10.1007/s43032-022-00896-0

**Published:** 2022-02-25

**Authors:** Wei Wei, Kouji Komatsu, Satoko Osuka, Tomohiko Murase, Bayasula Bayasula, Natsuki Nakanishi, Tomoko Nakamura, Maki Goto, Akira Iwase, Satoru Masubuchi, Hiroaki Kajiyama

**Affiliations:** 1 Department of Obstetrics and Gynecology, Nagoya University Graduate School of Medicine, 65 Tsurumai-Cho, Showa-Ku, Nagoya, Aichi 466-8550 Japan; 2 Department of Physiology, Aichi Medical University, 1-1 Yazakokarimata, Nagakute, Aichi 480-1195 Japan; 3 Department of Maternal and Perinatal Medicine, Nagoya University Hospital, 65 Tsurumai-Cho, Showa-Ku, Nagoya, Aichi 466-8550 Japan; 4 Bell Research Center for Reproductive Health and Cancer, Department of Obstetrics and Gynecology, Nagoya University Graduate School of Medicine, Nagoya, Aichi 466-8550 Japan; 5 Department of Obstetrics and Gynecology, Gunma University Graduate School of Medicine, 3-39-22 Showa-machi, Maebashi, 371-8511 Japan

**Keywords:** Primordial follicle, Tamoxifen, 17β-estradiol, Collagen type IV

## Abstract

Our previous study found that 17β-estradiol (E_2_) suppresses primordial follicle activation and growth in cultured mouse ovaries. In this study, we administered tamoxifen, an estrogen receptor antagonist, into the abdominal cavity of mice to clarify the relationship between primordial follicle activation and the physiological concentration of E_2_ in mouse ovaries. The results showed that tamoxifen promoted primordial follicle activation. Administration of tamoxifen promoted degradation of the extracellular matrix surrounding primordial follicles in the ovaries. Furthermore, tamoxifen decreased the expression of stefin A, an inhibitor of cathepsins that digest some proteins and extracellular matrix, in the ovaries. Mechanical stress produced by the extracellular matrix reportedly suppresses the activation of primordial follicles. The collective results show that tamoxifen can promote primordial follicle activation through the degradation of the extracellular matrix surrounding primordial follicles. Our results indicate that E_2_ suppresses primordial follicle activation in vivo and that tamoxifen may be useful as a therapeutic agent against infertility.

**Graphical abstract:**

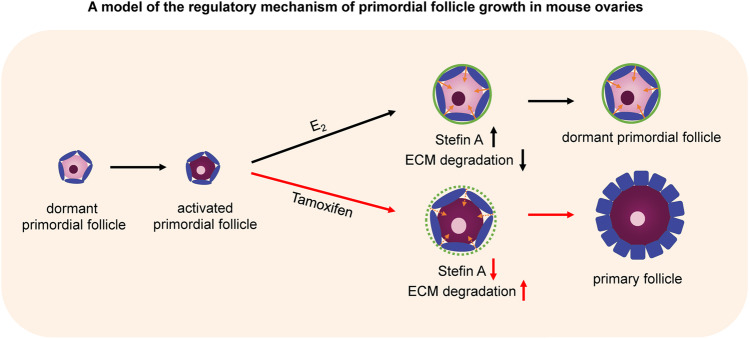

**Supplementary Information:**

The online version contains supplementary material available at 10.1007/s43032-022-00896-0.

## Introduction

Ovarian folliculogenesis starts with the recruitment of dormant primordial follicles into the growing follicle pool to form a primary, secondary, preantral follicle, and ultimately a preovulatory antral follicle [1]. It has been widely established that this precisely ordered process is regulated by the hypothalamic-pituitary–gonadal (HPG) axis. Gonadotropin-releasing hormone (GnRH), secreted by the hypothalamus, stimulates the production of pituitary gonadotropins follicle-stimulating hormone (FSH) and luteinizing hormone (LH), which play a predominant role in controlling the preovulatory stage of folliculogenesis, where a follicle is comprised of an antrum and subsequently successfully ovulate. However, earlier stages of folliculogenesis appear to be independent of pituitary gonadotropins [2, 3]. Most primordial follicles remain in a quiescent state, where dormant oocytes are arrested at the prophase of meiosis I, providing a reserve for continuous reproductive success [4, 5]. The activation or loss of primordial follicles is responsible for the irreversible decline in reproductive capacity [6]. Several intrinsic signaling pathways in oocytes have been implicated in the control of oocytes growth or dormancy. The phosphoinositide 3-kinase (PI3K)-serine/threonine kinase (AKT)–mammalian target of rapamycin (mTOR) pathway is considered to be the critical pathway that contributes to the activation and survival of primordial follicles, whereas the transcription factor forkhead boxO3 (FOXO3) and the phosphatase and tensin homolog deleted from chromosome 10 (PTEN) are essential for maintaining their quiescence [7–10]. In addition, several paracrine factors that balance the activation and suppression of primordial follicle growth have been identified. Growth and differentiation factor-9 (GDF-9), leukemia inhibitory factor (LIF), insulin-like growth factor-1 (IGF-1), bone morphogenic protein-4 (BMP-4), kit ligand (KL), and basic fibroblast growth factor (bFGF) are activators of the system [11–16], while anti-Müllerian hormone (AMH) is an inhibitor of the transition from primordial to primary [17]. Recently, some reports have shown that environmental factors, such as angiogenesis, hypoxia, and mechanical stress, could affect the maintenance of the dormant state of oocytes [18–20].

Previous studies have shown that 17β-estradiol (E_2_) inhibits oocyte cyst breakdown and primordial follicle formation in vitro and in vivo [4, 21]. Moreover, loss of estrogen receptor β (ESR2), the predominant estrogen receptor in the ovary, leads to primordial follicle activation (PFA) [22]. These results suggest that E_2_ also controls PFA. In fact, our previous research showed that E_2_, stefin A (STFA; also called cystatin A), and cathepsins control PFA and the growth of primordial follicles in mouse ovaries [23]. In this study, we attempted to promote PFA in mouse ovaries by peritoneal administration of tamoxifen, a competitive inhibitor of E_2_. Tamoxifen is a selective estrogen receptor modulator that prevents and treats estrogen receptor-positive breast cancer in pre-and postmenopausal women. Tamoxifen is also effective in mice and is widely used to induce Cre/loxP site-specific recombination systems for gene regulation [24]. Furthermore, we investigated the relationship between PFA during the estrous cycle and estradiol concentration in the serum and ovarian tissue to clarify the physiological mechanism of PFA.

## Materials and Methods

### Animals

This study was performed using experimental and care protocols approved by the Animal Experimental Committee of the Nagoya University Graduate School of Medicine in accordance with the relevant guidelines and regulations. C57BL/6 J female mice (10- to 20-week-old, Charles River Laboratories Japan, Inc., Kanagawa, Japan) were housed in an environmentally controlled room maintained at 23 ± 1 °C with a 12-h light/12-h dark photoperiod.

### Assessment of Estrous Cyclicity

The stages of the estrous cycle were determined by vaginal lavage. The vaginal smears were flushed by introducing 20-μl phosphate-buffered saline (PBS) through a pipette, and the fluid was then placed on a glass slide, allowing the smear to completely dry at room temperature. The smears were then stained using Giemsa solution (FUJIFILM Wako Pure Chemical Corp., Osaka, Japan) and observed under a light microscope (Carl Zeiss Co., Ltd., Tokyo, Japan). The estrous cycle stage was classified as proestrus, estrus, metestrus, and diestrus, based on the shape and density of the cells: nucleated epithelial cells, cornified epithelial cells, and leukocytes [25].

### Preparation of Ovarian Sections

Ovaries from each estrous stage were removed following the vaginal smear check and fixed with SUPER FIX (Kurabo, Osaka, Japan) at 4 °C overnight and then embedded in paraffin blocks. Ovaries were sectioned serially at 5-μm width and mounted on slides. Every tenth section was selected for staining. Approximately 20–28 sections per ovary were stained for immunohistochemistry.

### Tamoxifen Administration

Tamoxifen (Sigma-Aldrich Co. LLC, St. Louis, MO, USA) was injected into the abdominal cavity of mice. Tamoxifen was initially dissolved in 99.5% ethanol (100 mg/mL; FUJIFILM). Before injection, tamoxifen was diluted 10 × with sunflower seed oil (Sigma-Aldrich Co. LLC). An equal volume of sunflower seed oil containing 10% ethanol was injected as a control, according to the methods of a previous study [26]. The ovaries were removed 24 h after the tamoxifen injection. Left ovaries were used for immunohistochemistry, and the right ovaries were used for quantitative PCR.

### Immunohistochemistry

Briefly, after standard deparaffinization and rehydration, sections were immersed in citrate buffer (pH 6.0) and incubated at 95 °C for 20 min using a microwave for antigen retrieval. Sections were then incubated in 0.3% H_2_O_2_ for 20 min and then in blocking solution for 1 h at room temperature (20–25 °C) [27]. Next, the sections were incubated with anti-FOXO3a (1:2000 dilution, D19A7, Cell Signaling Technology, Danvers, MA, USA), anti-cystatin A (1:200 dilution, bs-4937R, Bioss Antibodies, Woburn, MA, USA), and anti-collagen type IV (1:500 dilution, ab19808, Abcam, Cambridge, UK) at 4 °C overnight. Rabbit immunoglobulin G (rabbit IgG, polyclonal–isotype control, ab37415; Abcam) was used as a negative control. Sections were then subjected to immunoperoxidase staining using the VECTASTAIN ABC Kit (Vector Laboratories, Burlingame, CA, USA) according to the standard protocol. Finally, all sections were counterstained with hematoxylin (FUJIFILM), dehydrated, and mounted.

Next, the sections were incubated with anti-FOXO3a (1:2000 dilution, D19A7, Cell Signaling Technology, Danvers, MA, USA), anti-cystatin A (1:200 dilution, bs-4937R, Bioss Antibodies, Woburn, MA, USA), and anti-collagen type IV (1:500 dilution, ab19808, Abcam, Cambridge, UK) at 4 °C overnight. Rabbit immunoglobulin G (rabbit IgG, polyclonal–isotype control, ab37415; Abcam) was used as a negative control. Sections were then subjected to immunoperoxidase staining using the VECTASTAIN ABC Kit (Vector Laboratories, Burlingame, CA, USA) according to the standard protocol. Finally, all sections were counterstained with hematoxylin (FUJIFILM), dehydrated, and mounted.

### Follicle Classification

The classification of follicles depends on the form of the granulosa cells. Follicles containing a single squamous granulosa cell layer were classified as primordial follicles; those containing a single cubical granulosa cell layer were classified as primary follicles; those containing multiple layers of granulosa cells and a follicular antrum were classified as antral follicles; those containing an oocyte with a pyknotic nucleus, vacuolated cytoplasm, and detached granulosa cells were classified as atretic follicles. Follicles were then classified into either activated or dormant follicles; FOXO3a localizes either in the nuclei of oocytes within dormant follicles or within the cytosol of oocytes in activated follicles. Only follicles containing an oocyte were counted, and atretic follicles were excluded.

### *Measurement of E*_*2*_

A pair of ovaries was removed from 10- to 20-week-old mice (five mice per group). Proteins were extracted using 300 μL RIPA lysis buffer (20–188; Merck, Burlington, MA, USA). Blood was extracted by terminal cardiac puncture from each mouse when sacrificed, and serum was separated by centrifugation at 3000 × *g* for 10 min at 4 °C. The ovaries and sera used in this experiment were obtained from the same mice. The E_2_ concentration in the ovaries and serum was determined using a chemiluminescent immunoassay (Estradiol II CalSet II, Elecsys, Cobas, Roche Diagnostics, Indianapolis, IN, USA). The intra-assay coefficient of variation was < 10%.

### Quantitative PCR

To confirm the effect of tamoxifen on the expression on STFA, total RNA was extracted from the right ovaries using the RNeasy Mini Kit (QIAGEN) following the manufacturer’s protocol. Reverse transcription (RT) reaction with 1 µg of total RNA was carried out using a first-strand cDNA synthesis kit (ReverTraAce-α-; Toyobo Co., Ltd., Osaka, Japan). The cDNA was diluted 1:10, and quantitative PCR (qPCR) was performed in 96-well, 0.2-mL thin-wall PCR plates using the Thermal Cycler Dice (Takara Bio Inc.). The real-time PCR mixture contained KOD SYBR qPCR Mix (Toyobo Co., Ltd.) (10 µL), primers (2 µM), and cDNA template (1 µg) in a total volume of 20 µL. The PCR protocol involved an initial incubation at 98 °C for 2 min, denaturation at 98 °C for 10 s, annealing at 60 °C for 10 s (45 cycles), and extension at 68 °C for 30 s. The primer sequences were STFA, 5′-GAGTCTTGGAGGTGTTTCAGAGG-3′ and 5′-TCCAGCGACGACTTGAGTTTTA-3′ (148 bp); and.

glyceraldehyde-3-phosphate dehydrogenase (Gapdh), 5′-ATGAATACGGCTACAGCAACAGG-3′, and 5′-CTGTTGCTCAGTGTCCTTGCTG-3′ (102 bp). We used Gapdh as an internal control. qPCR was performed in triplicate for all samples. Quantification was performed by calculating the ratio of the gene of interest to Gapdh mRNA using the comparative Ct method.

### Statistical Analyses

Statistical analyses were performed with GraphPad Prism software (version 8, San Diego, CA, USA) using one-way analysis of variance (ANOVA) and unpaired *t*-tests. Statistical significance was set at *P* < 0.05. The data are expressed as mean ± SD unless otherwise specified.

## Results

### Analysis of the Activation of Follicles During Estrous

**Fig. 1:**
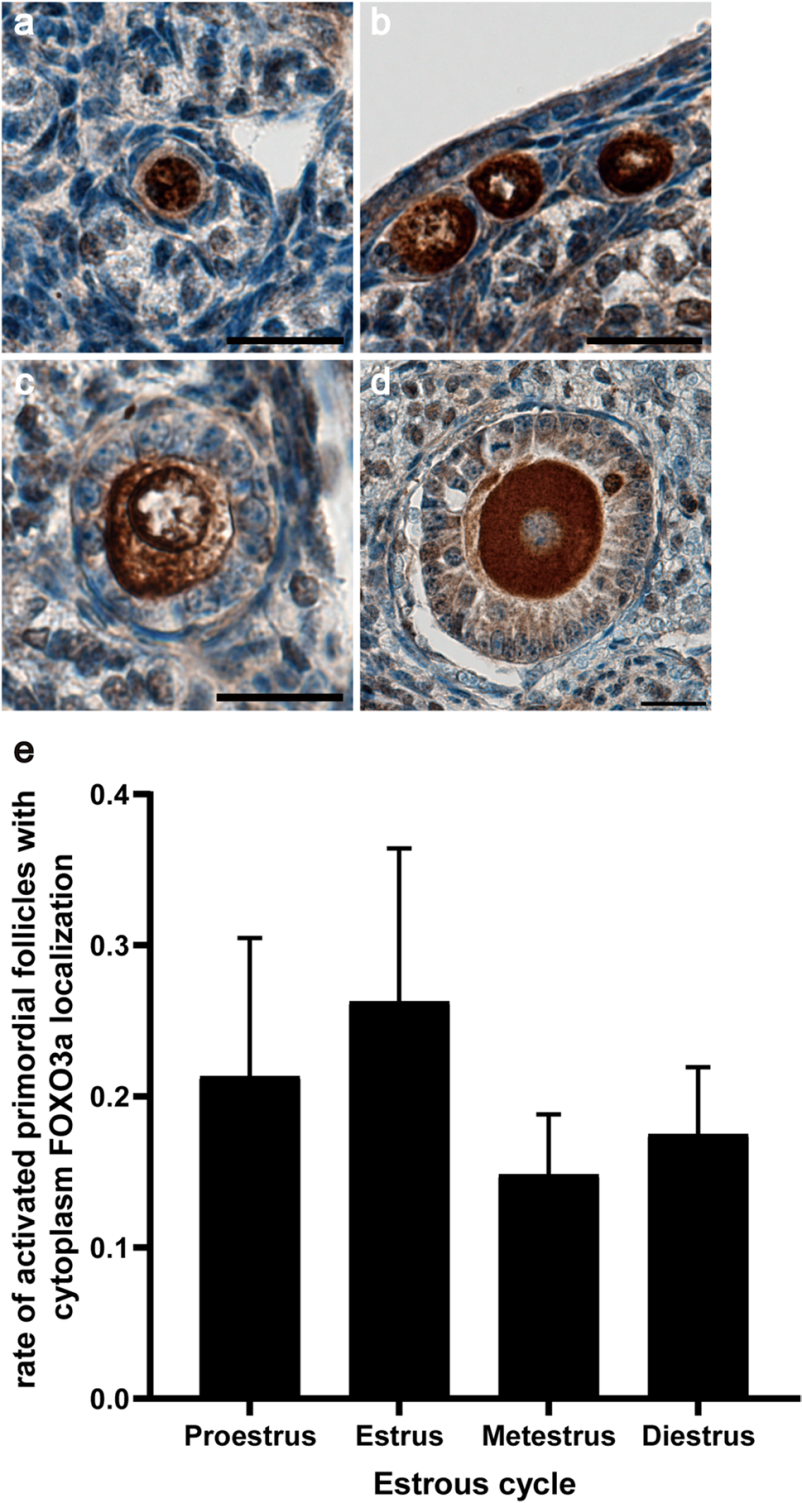
Analysis of the activation of follicles during estrous. **a**–**d** Localization of forkhead boxO3a (FOXO3a) in dormant (**a**) and activated (**b**) primordial follicles, primary follicles (**c**), secondary follicles (**d**) by immunohistochemistry. **e** Rate of activated primordial and primary follicles with cytoplasm FOXO3a localization in ovaries at each stage of the estrous cycle. Black bar: primordial follicle; gray bar: primary follicles. Mean ± SD (*n* = 3 for proestrus and metestrus, *n* = 4 for diestrus, and *n* = 5 for estrus). Scale bar 20 μm

Serial sections of ovaries were stained with an anti-FOXO3a antibody. FOXO3a localizes in the nuclei of oocytes in dormant follicles, whereas it localizes to the cytosol of oocytes in activated follicles. In primordial follicles, there were both dormant and activated follicles in the ovaries (Fig. 1a, b). In primary and secondary follicles, FOXO3a was localized in the oocyte cytoplasm (Fig. 1c, d). The number of activated primordial follicles in each ovary during the four stages of the estrous cycle (proestrus, estrus, metestrus, and diestrus) was evaluated (Supplementary Table 1). There were no significant differences in the rate of activated primordial and primary follicles during the estrous cycle (Fig. 1e; primordial follicle: proestrus: 0.21 ± 0.09, estrus: 0.26 ± 0.10, metestrus: 0.15 ± 0.04, diestrus: 0.18 ± 0.04; primary follicle: proestrus: 0.44 ± 0.12, estrus: 0.46 ± 0.13, metestrus: 0.38 ± 0.06, diestrus: 0.40 ± 0.1).

### Administration with Tamoxifen

**Fig. 2:**
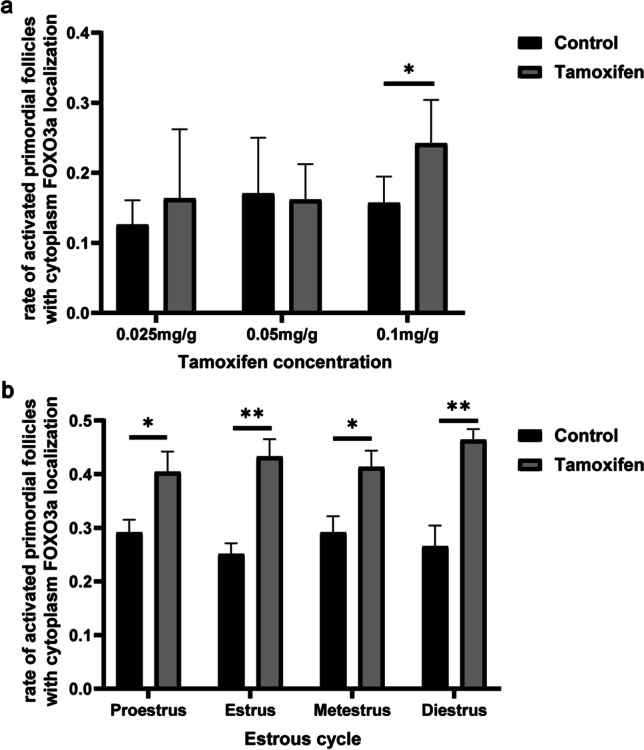
Rate of activated follicles in ovaries treated with tamoxifen. **a** Rate of activated primordial follicles with cytoplasm forkhead boxO3a (FOXO3a) localization in ovaries treated with 0.025 mg/g body weight, 0.05 mg/g body weight, and 0.1 mg/g body weight tamoxifen. Black bar: control; gray bar: tamoxifen. Mean ± SD (*n* = 4 for 0.025 mg/g body weight and 0.05 mg/g body weight, *n* = 5 for 0.1 mg/g body weight control, and *n* = 6 for 0.1 mg/g body weight tamoxifen). *P < 0.05. **b** Rate of activated primordial follicles with cytoplasm FOXO3a localization in four stages of the estrous cycle after administration of 0.1 mg/g body weight tamoxifen. Black bar: control; gray bar: tamoxifen. Mean ± SD (*n* = 5). *P < 0.05 and **P < 0.01

To confirm whether inhibition of the E_2_ effect promotes PFA in vivo, we injected tamoxifen into the abdominal cavity of metestrus mice and found that the rate of activated primordial follicles in the metestrus was the lowest during the estrous cycle (*P* = 0.341, Fig. 1e). Tamoxifen was injected at 0.025, 0.05, and 0.1 mg/g body weight. After 24 h from administration, the rate of activated primordial follicles in 0.1 mg/g body weight tamoxifen-treated ovaries were significantly increased (Fig. 2a and Supplementary Table 2; 0.025 mg/g body weight control: 0.13 ± 0.03, 0.025 mg/g body weight tamoxifen: 0.16 ± 0.09, 0.05 mg/g body weight control:0.17 ± 0.07, 0.05 mg/g body weight tamoxifen: 0.16 ± 0.04, 0.1 mg/g body weight control: 0.16 ± 0.03, 0.1 mg/g body weight tamoxifen: 0.24 ± 0.03). Tamoxifen was then administered to 10 to 20-week-old mice of each estrous cycle stage at 0.1 mg/g body weight. Tamoxifen administration increased the rate of activated primordial follicles in all estrous cycle stages, and there were no significant differences between each estrous cycle stage (Fig. 2b and Supplementary Table 3; proestrus: control: 0.29 ± 0.05, tamoxifen: 0.40 ± 0.08; estrus: control: 0.25 ± 0.04, tamoxifen: 0.43 ± 0.07; metestrus: control: 0.29 ± 0.06, tamoxifen: 0.41 ± 0.06; diestrus: control: 0.27 ± 0.08, tamoxifen: 0.46 ± 0.04).

### *Measurement of E*_*2*_* Concentration at Each Stage of the Estrous Cycle in the Serum and the Ovary*

**Fig. 3:**
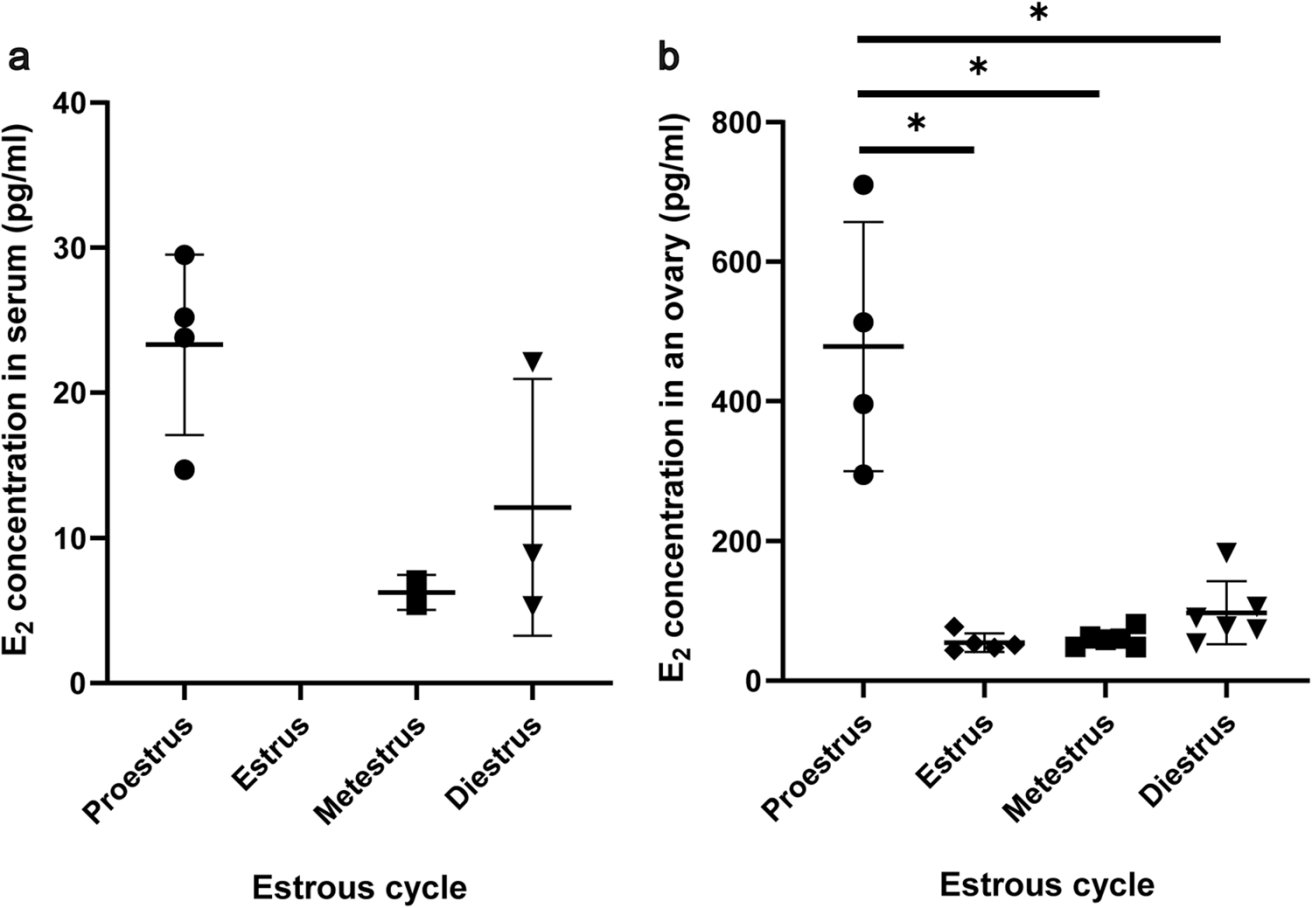
E_2_ levels at each stage of the estrous cycle in the serum and ovary. **a** E_2_ levels at each stage of the estrous cycle in the serum. E_2_ levels in the serum of estrus mice and some metestrus and diestrus mice were undetectable (< 5.0 pg/mL). Mean ± SD (*n* = 4 for proestrus, *n* = 2 for metestrus, *n* = 3 for diestrus). **b** E_2_ levels at each stage of the estrous cycle in the ovary. Mean ± SD (*n* = 4 for proestrus, *n* = 5 for estrus, *n* = 6 for metestrus and diestrus). *P < 0.05

We assessed the E_2_ concentration in the serum and ovaries during the estrous cycle (Fig. 3 and Table 1). E_2_ levels in the serum and ovaries fluctuated during the estrous cycle (Fig. 3a and 3b). In the ovary, E_2_ concentration is significantly higher during proestrus (478.5 ± 154.3 pg/mL) than all other stages and decline during estrus (54.58 ± 11.80 pg/mL) and then gradually rises again during metestrus and diestrus (Fig. 3b; metestrus: 59.97 ± 11.18 pg/mL, diestrus: 97.4 ± 41.48 pg/mL, *P* = 0.09). This result is consistent with the trend in E_2_ serum concentration (Fig. 3a and Table 1). The E_2_ concentration level in the serum of some mice was too low to be detected (< 5.0 pg/mL, Table 1) and therefore classified as undetectable data during the statistical processing. These results indicated that there was no relationship between PFA and the change in E_2_ concentration in the serum and ovaries during the estrous cycle (Fig. 1f, 2b, and 3).

**Table 1:** E_2_ concentration in the serum and ovary

	Proestrus		Estrus		Metestrus		Diestrus	
Mouse no	Serum (pg/mL)	Ovary (pg/mL)	Serum (pg/mL)	Ovary (pg/mL)	Serum (pg/mL)	Ovary (pg/mL)	Serum (pg/mL)	Ovary (pg/mL)
1	14.7	295	< 5.0	47.2	< 5.0	63.8	< 5.0	106
2	25.2	396	< 5.0	51	7.1	60.2	22.1	90.4
3	23.8	513	< 5.0	53.7	< 5.0	81.2	< 5.0	53.5
4	29.5	710	< 5.0	77.2	5.4	48.4	5.3	78
5			< 5.0	43.8	< 5.0	58.4	< 5.0	73.5
6					< 5.0	47.8	8.9	183
Average SD	23.3 5.39	478.5 154.3		54.58^a^ 11.8		59.97^a^ 11.18	12.1 7.22	97.4^a^ 41.48

a: < 0.05 vs. proestrus

### Analysis of PFA in the Local Area

**Fig. 4:**
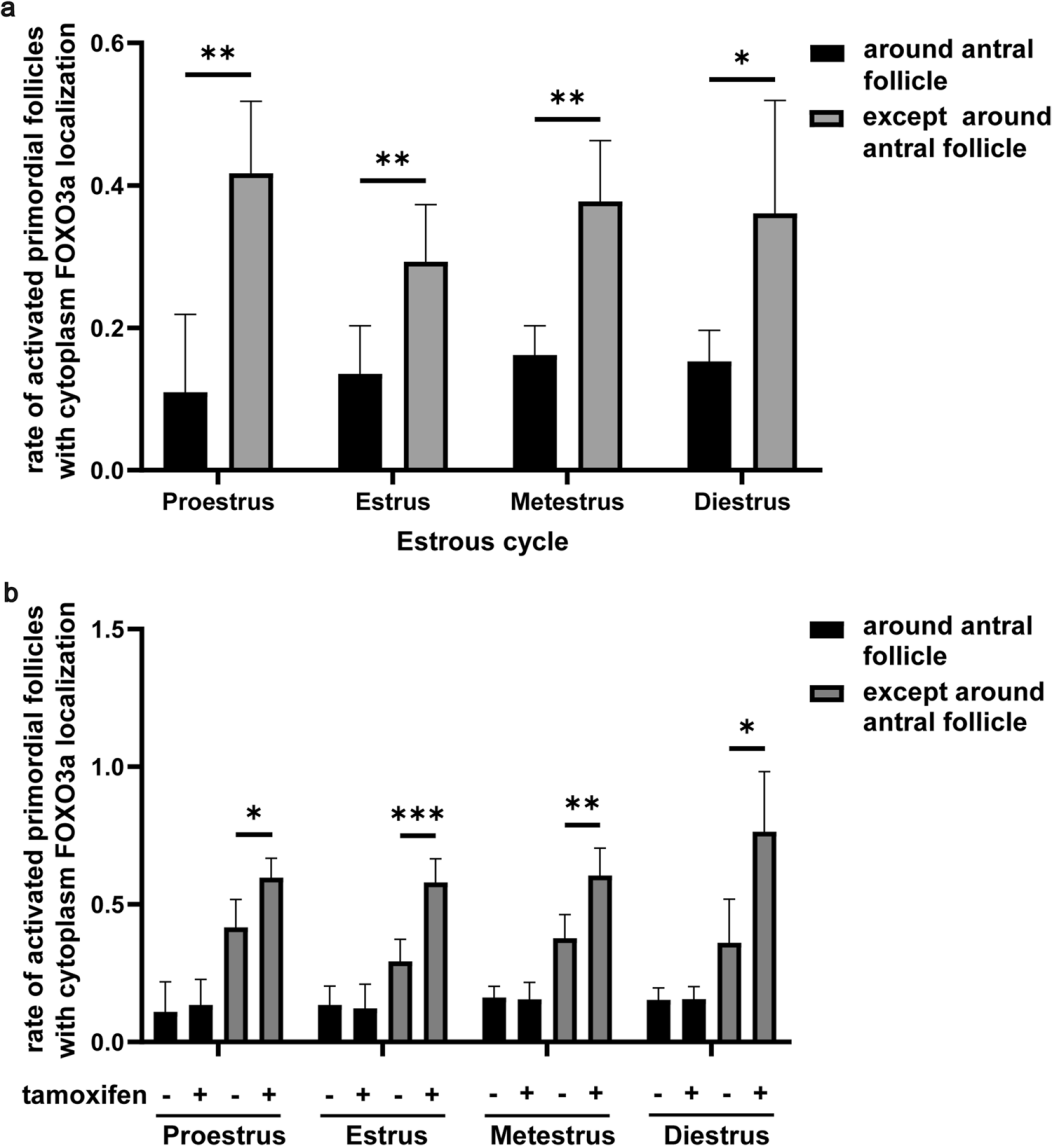
Rate of activated primordial follicles in the local area of the ovary. **a** Comparison of the rate of activated primordial follicles with cytoplasm forkhead boxO3a (FOXO3a) localization around the antral follicles and the rest. Mean ± SD (*n* = 5). *P < 0.05, **P < 0.01. **b** Rate of activated primordial follicles with cytoplasm FOXO3a localization around the antral follicles or not in ovaries treated with tamoxifen. Mean ± SD (*n* = 5). *P < 0.05, **P < 0.01, and ***P < 0.001

Estrogen is produced by the granulosa cells of late preantral follicles and reaches the highest intrafollicular levels in the preovulatory follicles [28]. To clarify whether the E_2_ concentration in the local area of the ovary affects PFA, we evaluated the number of primordial follicles within 50 μm around the antral follicles. The results showed that the rate of activated primordial follicles was lower than that of the rest of the ovaries (Fig. 4a and Supplementary Table 4; proestrus: around antral follicles: 0.11 ± 0.1, except around antral follicles: 0.42 ± 0.09; estrus: around antral follicles: 0.14 ± 0.06, except around antral follicles: 0.29 ± 0.07; metestrus: around antral follicles: 0.16 ± 0.04, except around antral follicles: 0.38 ± 0.08; diestrus: around antral follicles: 0.15 ± 0.04, except around antral follicles: 0.36 ± 0.14). After tamoxifen administration, there was no significant change in the rate of activated primordial follicles around antral follicles in all stages of the estrous cycle (Fig. 4b and Supplementary Table 4; proestrus: control: 0.11 ± 0.1, tamoxifen: 0.13 ± 0.08; estrus: control: 0.14 ± 0.06, tamoxifen: 0.12 ± 0.08; metestrus: control: 0.16 ± 0.04, tamoxifen: 0.15 ± 0.06; diestrus: control: 0.15 ± 0.04, tamoxifen: 0.16 ± 0.04). These results showed that tamoxifen promoted PFA except for the primordial follicles around the antral follicles.

### Effect of Tamoxifen on STFA Expression and Analysis of the Digestion of Extracellular Matrix Around Primordial Follicles

**Fig. 5:**
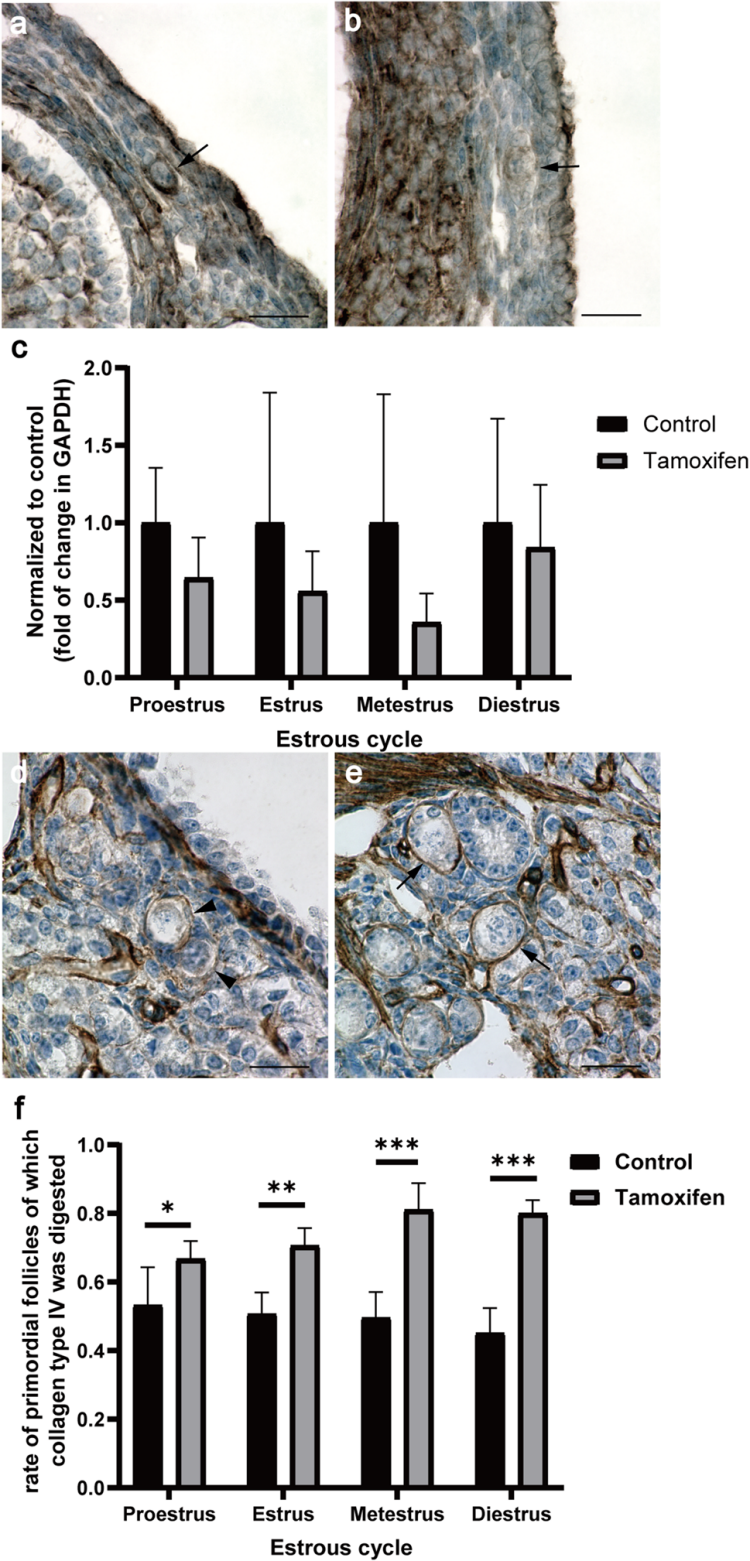
Stefin A (STFA; Cystatin A) in primordial follicles and digestion of collagen type IV in ovaries treated with tamoxifen. **a**, **b** Images of 12-week-old mouse ovaries stained with anti-cystatin A antibody. STFA was strongly expressed in most primordial follicles (**a**) but was weakly expressed in some (**b**). Arrows indicated primordial follicles. Scale bar: 20 μm. **c** Effect of tamoxifen on the expression of STFA. STFA mRNA expression levels in four stages of the estrous cycle were measured by qPCR after treatment with 0.1 mg/g body weight tamoxifen. Black bar: control; gray bar: tamoxifen. The results are expressed as mean ± SD (*n* = 5). **d**, **e** Images of 10 to 20-week-old mouse ovaries stained with anti-collagen type IV antibody. Arrowheads indicated primordial follicles around which collagen type IV was completely or partially digested. An arrow indicated a primordial follicle that was completely surrounded by collagen type IV. Scale bar: 20 μm. **f** Rate of primordial follicles around which collagen type IV was digested during estrous cycles. Mean ± SD (*n* = 5). *P < 0.05, **P < 0.01, and ***P < 0.001

Our previous research showed that E_2_ regulates the expression of STFA and controls the activation and growth of primordial follicles through cathepsin-mediated digestion of the extracellular matrix (ECM) around primordial follicles [23]. STFA was localized in the oocytes and granulosa cells of primordial follicles in 12-week-old mouse ovaries (Fig. 5a and b). The expression levels of STFA in the primordial follicles varied. Most primordial follicles strongly expressed STFA (Fig. 5a), but their expression levels were low in some primordial follicles (Fig. 5b). We further studied the effect of tamoxifen on the expression of STFA in ovaries. Tamoxifen tended to decrease the expression of STFA in all estrous cycle stages, with the following values: proestrus: control: 1.00 ± 0.32, tamoxifen: 0.65 ± 0.23, *P* = 0.108; estrus: control: 1.00 ± 0.75, tamoxifen: 0.56 ± 0.23, *P* = 0.421; metestrus: control: 1.00 ± 0.74, tamoxifen: 0.36 ± 0.17, *P* = 0.31; diestrus: control: 1.00 ± 0.60, tamoxifen: 0.84 ± 0.36, *P* = 0.661 (Fig. 5c).

To confirm ECM digestion around the primordial follicles, we observed collagen type IV in the ovaries of mice treated with tamoxifen (Fig. 5d and e). The rate of primordial follicles containing some surrounding digested collagen type IV was significantly increased in tamoxifen-treated ovaries, with no significant difference between the four stages of the estrous cycle (Fig. 5f and Supplementary Table 5; proestrus: control: 0.53 ± 0.1, tamoxifen: 0.67 ± 0.05; estrus: control: 0.51 ± 0.06, tamoxifen: 0.12 ± 0.08; metestrus: control: 0.5 ± 0.07, tamoxifen: 0.81 ± 0.07; diestrus: control: 0.45 ± 0.07, tamoxifen: 0.8 ± 0.03). This result correlated with the increased rate of activated primordial follicles in ovaries treated with tamoxifen (Fig. 2b and 5f).

## Discussion

In this study, we confirmed that tamoxifen administration promoted PFA, consistent with our findings in the ovarian tissue culture experiments; thus, E_2_ also inhibited PFA in vivo (Fig. 2). However, there was no correlation between the concentration of E_2_ in the ovaries and PFA (Fig. 1f and 3). Therefore, we speculated that the local concentration of E_2_ might be important for the control of PFA. In our previous report, the activation and growth of primordial follicles were suppressed by E_2_ in ovaries from 4-day postnatal mice, in which primordial and primary follicles exist [23]. E_2_ is mainly secreted by antral follicles, so the concentration in serum and ovarian tissues is correlated during the estrous cycle (Fig. 3 and Table 1). However, antral follicles are not the only tissue that secretes E_2_ in the ovaries. The expression and activity of aromatase have been observed in the granulosa cells of primary follicles [29, 30]. These results indicate that the concentration of E_2_ in the ovary may be nonuniform in ovaries and that PFA depends on the local concentration of E_2_ in the ovaries. Tamoxifen promoted PFA in mouse ovaries but not around antral follicles (Fig. 4). Therefore, we need to clarify the concentration profile of E_2_ in the ovaries to understand the physiological mechanisms controlling PFA.

As the primordial follicles around antral follicles are not affected by tamoxifen administration, there are likely suppressive factors other than estrogen. AMH is expressed explicitly in the granulosa cells of growing follicles and has been identified as an inhibitor of primordial follicle recruitment and the initiation of primordial follicle growth [31, 32]. In addition, chemoattractive cytokine stromal-derived factor-1 (SDF1) has been suggested to play a vital role in inhibiting follicle activation in an autocrine manner [33]. These inhibitory factors and E_2_ are considered to work together to maintain the quiescent state of the primordial follicles. It is possible that peritoneally administered tamoxifen did not spread to the entire ovary. However, this is unlikely because genetically modified mice have been produced by administering tamoxifen at the same concentration (0.1 mg/g body weight) or lower [26, 34].

To trace primordial follicle growth after PFA induced by tamoxifen administration, we enumerated the number of primordial, primary, and secondary follicles 1 to 3 weeks after tamoxifen administration. No significant changes were observed (data not shown). It is assumed that the primordial follicles activated by tamoxifen administration may have returned to a dormant state once tamoxifen had worn off. To promote the development of primordial follicles into primary follicles, a new administration method, such as multiple doses of tamoxifen or a combination of tamoxifen and other factors, should be developed.

The ECM is known to regulate gene expression, intracellular signaling, and structural remodeling, consequently influencing cell proliferation and differentiation. Collagens are some of the main structural proteins of the extracellular matrix that provide tensile strength and limit the distensibility of tissues [35]. Primordial follicles are enclosed in the collagen-rich ovarian cortex, which is a relatively rigid physical environment compared to the medulla comprising growing follicles [36]. The ECM surrounding primordial follicles produces mechanical stress and maintains the dormant state of primordial follicles [19]. STFA has been shown to control the growth of primordial follicles through cathepsin-mediated digestion of ECM around primordial follicles under the regulation of E_2_ in cultured ovaries [23]. Our data showed that STFA was expressed in primordial follicles and that tamoxifen administration induced the degradation of collagen type IV around primordial follicles, leading to the activation of primordial follicles (Fig. 5). qPCR results revealed no significant difference in STFA expression after tamoxifen treatment as we used the whole ovary, including mRNAs expressed in other tissues except primordial follicles (Fig. 5c). However, tamoxifen tended to decrease the expression of STFA, especially in the ovaries of the proestrus cycle (*P* = 0.108). As E_2_ concentration is the highest during proestrus, the effect of tamoxifen may be clear. Therefore, we postulate that tamoxifen suppresses STFA expression in vivo. These results suggest that E_2_ regulates the expression of stefin A and controls the degradation of collagen type IV around primordial follicles, thereby repressing PFA in vivo.

In conclusion, we demonstrated that this estrogen-receptor antagonist activates primordial follicles in vivo and revealed that E_2_ is a critical physiological factor for regulating PFA by controlling the digestion of ECM around primordial follicles. Future studies should investigate the concentration profile of E_2_ in the ovaries to clarify the physiological mechanisms controlling PFA. Tamoxifen is already used as an anticancer drug, and our results indicate the possibility that tamoxifen may be useful as a therapeutic agent for infertility.

## Supplementary Information

Below is the link to the electronic supplementary material.

Supplementary file1 (PDF 138 KB)

## Data Availability

Not applicable.

## Code Availability

Prism software (Prism 8; GraphPad Software, San Diego, CA, USA).
