# Supplementary material for: Tamoxifen Activates Dormant Primordial Follicles in Mouse Ovaries
Source: Reprod Sci. 2022 Feb 25;29(12):3404–12. doi: 10.1007/s43032-022-00896-0 (PMC9734234; doi:10.1007/s43032-022-00896-0)
Supplement: Supplementary file 1 — Supplementary file1 (PDF 138 KB) [file 43032_2022_896_MOESM1_ESM.pdf]

Supplementary Table 1 Number of FOXO3a nuclei and cytoplasmic localization in primordial follicles during the four stages of the estrous cycle

	Proestrus		Estrus		Metestrus		Diestrus	
mouse no.	nuclei	cytoplasm	nuclei	cytoplasm	nuclei	cytoplasm	nuclei	cytoplasm
1	124	49	278	93	285	73	268	60
2	131	12	274	68	243	31	214	31
3	144	54	130	21	191	28	53	9
4			67	27			115	37
5			133	104				

Supplementary Table 2 Number of FOXO3a nuclei and cytoplasmic localization in primordial follicles in ovaries treated with tamoxifen

	0.025mg/g Control		0.025mg/g Tamoxifen		0.05mg/g Control		0.05mg/g Tamoxifen		0.1mg/g Control		0.1mg/g Tamoxifen	
mouse no.	nuclei	cytoplasm	nuclei	cytoplasm	nuclei	cytoplasm	nuclei	cytoplasm	nuclei	cytoplasm	nuclei	cytoplasm
1	87	16	29	2	67	24	33	7	17	2	29	11
2	81	10	16	6	38	10	63	18	40	10	79	28
3	33	6	57	16	39	4	80	14	76	14	62	29
4	87	8	131	14	84	11	81	9	75	12	94	27
5									70	16	67	21
6											39	6

Supplementary Table 3 Number of FOXO3a nuclei and cytoplasmic localization in primordial follicles and primary follicles during the estrous cycle following administration of 0.1 mg/g tamoxifen

	Proestrus Control		Proestrus Tamoxifen		Estrus Control		Estrus Tamoxifen		Metestrus Control		Metestrus Tamoxifen		Diestrus Control		Diestrus Tamoxifen	
mouse no.	nuclei	cytoplasm	nuclei	cytoplasm	nuclei	cytoplasm	nuclei	cytoplasm	nuclei	cytoplasm	nuclei	cytoplasm	nuclei	cytoplasm	nuclei	cytoplasm
1	44	26	81	49	10	3	32	15	52	21	45	31	101	64	70	61
2	47	17	66	31	120	32	63	49	123	46	68	77	88	41	47	48
3	109	34	51	33	97	47	1	1	61	41	67	43	179	49	22	23
4	14	5	109	68	175	59	95	92	111	31	98	55	26	8	77	58
5	116	54	29	35	124	38	65	46	134	51	38	23	97	20	48	33

Supplementary Table 4 Number of FOXO3a nuclei and cytoplasmic localization in primordial follicles around antral follicles in ovaries treated with tamoxifen

tamoxifen (-)	Proestrus aroud antral follicle		Proestrus except aroud antral follicle		Estrus aroud antral follicle		Estrus except aroud antral follicle		Metestrus aroud antral follicle		Metestrus except aroud antral follicle		Diestrus aroud antral follicle		Diestrus except aroud antral follicle	
mouse no.	nuclei	cytoplasm	nuclei	cytoplasm	nuclei	cytoplasm	nuclei	cytoplasm	nuclei	cytoplasm	nuclei	cytoplasm	nuclei	cytoplasm	nuclei	cytoplasm
1	22	9	22	17	6	2	4	1	34	9	18	12	51	10	50	54
2	21	2	26	15	47	5	73	27	53	8	70	38	60	9	28	32
3	40	5	69	29	42	7	55	40	27	5	34	36	69	7	110	42
4	7	0	7	5	41	4	134	55	54	7	57	24	19	5	7	3
5	79	5	37	49	37	4	87	34	57	14	77	37	59	12	38	8

tamoxifen (+)	Proestrus aroud antral follicle		Proestrus except aroud antral follicle		Estrus aroud antral follicle		Estrus except aroud antral follicle		Metestrus aroud antral follicle		Metestrus except aroud antral follicle		Diestrus aroud antral follicle		Diestrus except aroud antral follicle	
mouse no.	nuclei	cytoplasm	nuclei	cytoplasm	nuclei	cytoplasm	nuclei	cytoplasm	nuclei	cytoplasm	nuclei	cytoplasm	Nuclei	cytoplasm	nuclei	cytoplasm
1	56	14	25	35	20	3	12	12	23	2	22	29	62	11	8	50
2	41	3	25	28	47	12	16	37	27	9	41	68	35	5	12	43
3	26	3	25	30	0	0	1	1	56	9	11	34	23	7	0	16
4	69	3	40	65	31	8	64	84	50	10	48	45	39	7	38	51
5	17	6	12	29	40	3	25	43	25	4	13	19	22	3	26	30

Supplementary Table 5 Number of primordial follicles around which collagen type IV was digested during the estrous cycles

	Proestrus Control		Proestrus Tamoxifen		Estrus Control		Estrus Tamoxifen		Metestrus Control		Metestrus Tamoxifen		Diestrus Control		Diestrus Tamoxifen	
mouse no.	digested	undigested	digested	undigested	digested	undigested	digested	undigested	digested	undigested	digested	undigested	digested	undigested	digested	undigested
1	32	37	86	48	11	16	48	19	24	39	67	22	55	104	100	28
2	25	39	84	44	103	91	85	43	85	70	107	43	46	68	106	33
3	80	45	64	38	82	70	1	2	50	45	163	17	105	96	35	6
4	22	20	122	39	101	106	151	44	62	72	161	31	26	28	126	36
5	94	52	61	32	79	60	61	30	96	75	65	12	52	52	67	14
